# Dietary Supplementation with Gotu Kola (*Centella asiatica*) Extract Enhanced Innate Immune Responses, Modulated Immune-Related Gene Expression, and Improved Gut Microbiota in Giant Freshwater Prawn (*Macrobrachium rosenbergii*)

**DOI:** 10.3390/ani15172507

**Published:** 2025-08-26

**Authors:** Phanupong Changtor, Donlaya Pinmuang, Channarong Nasalingkhan, Nonglak Yimtragool

**Affiliations:** 1 Department of Biology, Faculty of Science, Naresuan University, Phitsanulok 65000, Thailand; phanupongc@nu.ac.th (P.C.); donlayap63@nu.ac.th (D.P.); channarongn60@nu.ac.th (C.N.); 2 Center of Excellence for Innovation and Technology for Detection and Advanced Materials (ITDAM), Naresuan University, Phitsanulok 65000, Thailand; 3 Center of Excellence for Biodiversity, Faculty of Science, Naresuan University, Phitsanulok 65000, Thailand

**Keywords:** aquaculture, *C. asiatica*, feed additives, immune response, *M. rosenbergii*

## Abstract

The giant freshwater prawn (*Macrobrachium rosenbergii*) has economic significance in the aquatic industry, but production is impacted by infectious diseases induced by various pathogens. Herein, we investigated the impact of adding feed additives to the diet of *M*. *rosenbergii* to promote health. Diets were formulated to contain different levels of *Centella asiatica* (L.) Urb. crude extracts (1, 5, and 10 g kg^−1^), with growth performance and innate immune parameters assessed after 28 days of feeding. Real-time quantitative PCR (RT-qPCR) was employed to determine the mRNA levels of *serine proteinase inhibitor* (*SPI*) and *alpha-2 macroglobulin* (*Mr-2α2M*) from 12 h to 28 days of feeding. Prawns feeding at 5 and 10 g kg^−1^ showed statistically significant differences in specific growth rate, lysozyme assay, and phenoloxidase activity. The expression levels of all the immune-related genes studied were significantly upregulated in prawns fed with supplemented diets compared to the control group. Findings revealed that the observed upregulations varied in response to alterations in feeding time and concentration. Furthermore, 16S rRNA analysis showed that the supplemented diets at 10 g kg^−1^ supplementation increased beneficial bacteria (*Lactococcus* sp.) and reduced pathogenic taxa (e.g., *Candidatus Hepatoplasma*, *Flavobacteriaceae*, *Weeksellaceae*, *Thiothrix* sp.).

## Simple Summary

Infectious diseases represent a major obstacle to the sustainability and profitability of giant freshwater prawn aquaculture. In this study, we evaluated the potential of gotu kola crude extract as a dietary supplement to improve prawn health. Experimental diets were prepared by adding *C. asiatica* extract at concentrations of 1, 5, and 10 g kg^−1^ and administered over a 28-day period. The impact of these diets on prawn growth, innate immune responses, and gut microbiota was assessed. Quantitative real-time PCR was used to measure the expression of immune-related genes, including *serine proteinase inhibitor* and *alpha-2-macroglobulin*, at multiple time points post-feeding. Notably, the highest supplementation level (10 g kg^−1^) led to enhanced expression of immune genes, improved gut microbial balance by increasing beneficial bacteria and reducing pathogenic bacteria, and supported better overall innate immune parameters. These findings highlight the potential of gotu kola crude extract as a natural immunostimulant and health-promoting agent in aquaculture, offering a promising strategy to enhance disease resistance and sustainability in prawn farming.

## 1. Introduction

The giant freshwater prawn (*Macrobrachium rosenbergii*) is a significant economic aquatics animal for food production in several countries [1], with demand and cultivation increasing, driven primarily by the expanding human population [2]. However, aquaculture frequently faces challenges related to low survival rates of animals due to the intensive farming system [3]. The large numbers of animals in intensive farming programs is a key factor contributing to poor pond microenvironments. These conditions may increase the stress of shrimp, decreasing their health and making them more susceptible to pathogen infection. Pond preparation, stocking, and farming management are essential factors that influence prawn productivity [4]. The misuse of antibiotics is a significant concern throughout aquaculture; however, this issue is particularly acute in crustacean farming, especially for shrimp. Frequent bacterial outbreaks and a lack of approved therapeutic agents for invertebrates often lead to excessive and unregulated antibiotic use. Consequently, these practices not only contribute to the emergence of antibiotic-resistant bacteria but also result in the accumulation of harmful residues in both the environment and the food chain [5]. Numerous biological techniques have been reported for shrimp farm management with the aim of reducing antibiotic use, including vaccination [6], probiotics, and beneficial microbes [7]. Among these, the use of feed additives derived from medicinal plants represents a promising alternative to improving shrimp health in the production system.

Gotu kola (*Centella asiatica* (L.) Urban.) is a creeping perennial herb belonging to the family Apiaceae (formerly Umbelliferae) and native to Asia, South Africa, and Eastern South America [8,9]. This plant is abundant in phytochemicals such as asiaticoside, madecassoside, and brahmoside [10,11]. *C. asiatica* has demonstrated antifungal and antibacterial [12], antioxidant, and anti-inflammatory activities in human cells [13]. In previous studies, an aqueous extract of *C. asiatica* at concentration of 31.25 μg/mL showed inhibition of the growth of *Flavobacterium columnare*, a bacterial pathogen that causes columnaris disease in Nile tilapia (*Oreochromis niloticus*). Fish mortality rates in the bath treatments were reduced for experimentally induced columnaris disease [14]. Previous studies have reported that supplementing a fish diet with 5 and 10 g/kg of *C. asiatica* powder improved serum lysozyme activity, peroxidase activities, alternative complement, phagocytosis, and respiratory burst activity [15]. A diet with methanolic *C. asiatica* extracts at 0.79 and 12.50 mg/mL inhibited *Vibrio alginolyticus* infection in white leg shrimp (*Litopenaeus vannamei*) [16].

Crustaceans are invertebrates that lack a vertebrate-like adaptive immune system and do not have a specific immune response against infectious agents [17]. However, they have efficient innate immunity consisting of physical barriers and hemostatic mechanisms with cellular and humoral components [17,18]. *Serine protease inhibitor* (*SPI*) is an important protein defense against pathogenic microorganisms, regulating proteolytic activity. *SPI* catalyzes the conversion of inactive proPO to phenoloxidase (PO), a crucial enzyme in the defense system. *Alpha-2 macroglobulin* (*Mr-2α2M*) is a multifunctional protein and is one of the most important non-specific protease inhibitor proteases in the immune system [19]. It functions as a broad-spectrum trap, physically encapsulating and inactivating proteases from pathogens. The complex formed between *Mr-2α2M* and the protease is then recognized and removed from circulation via phagocytosis. This mechanism helps to control pathogen infection and also plays a crucial role in regulating the blood clotting and proPO systems [20]. Understanding the immune mechanisms involved in gene expression and enzyme activities is essential, particularly following supplementation of *C. asiatica* in the giant freshwater prawn, which has not been intensively studied.

Therefore, this study aimed to investigate the impact of supplementing *C. asiatica* extract at different levels on the growth, immunological parameters, immune-related gene expression, and intestine microbiota of giant freshwater prawns. Our results can be used to develop a more sustainable and health-conscious approach to environmentally friendly prawn farming, ultimately contributing to safer products for consumers.

## 2. Materials and Methods

### 2.1. Ethics Statement

The animal welfare and care protocol for these experiments, number NU-AQ640201, was approved by the Ethics Committee on the Care and Use of Laboratory Animals at Naresuan University.

### 2.2. Preparation of C. Asiatica Extract and Experiment Diets

Gotu kola (*Centella asiatica* (L.) Urban.) was purchased from a local market in Phitsanulok, Thailand. The leaves and stems were washed and then dehydrated at 55 °C for 3 days before being ground into a powder. The extraction was performed using 95% ethanol at a *C. asiatica* powder-to-solvent ratio of 1:10 for 6 days, with the ethanol replaced every 2 days. The total extracted solution was pooled and filtered. The filtered solution was evaporated at 55 °C and dried using the freeze-drying method. The amounts of the four major triterpenoids in the *C. asiatica* crude extract, including asiatic acid, asiaticoside, madecassic acid, and madecassoside, were determined using high-performance liquid chromatography (HPLC) according to Kamol et al. (2023) [9]. The centelloside composition of *C. asiatica* extracted using ethanol included madecassoside 0.93 ± 0.26% DW (%DW: g/100 g), madecassic acid 0.18 ± 0.06% DW, asiatic acid 0.20 ± 0.05% DW, and asiaticoside 0.88 ± 0.13% DW (Supplementary Table S1). Four experimental diets were formulated by mixing a commercial diet with Thai banana (*Musa acuminata* × *Musa balbisiana*), which was used as a feed binder (containing 32.84% crude protein, 3.08% crude lipid, 10.73% ash, 9.34% moisture, 2.68% fiber, and 41.33% carbohydrate) and supplemented with dried crude extract of *C. asiatica* at concentrations of 1, 5, and 10 g/kg of feed. This solution was then uniformly sprayed onto the commercial diet, and the mixture was thoroughly homogenized using a kitchen mixer for 10 min to ensure even distribution of the extract. The prepared diets were then air-dried at room temperature for 24 h to evaporate the ethanol before being stored at 4 °C.

### 2.3. Prawn Cultivation and Tissue Collection

Giant freshwater prawns (P30: post-larval stage) were obtained from a commercial farm in Phitsanulok, Thailand. The prawns were shipped to a laboratory in the Department of Biology, Naresuan University and quarantined in a 500 L plastic tank containing aerated freshwater at room temperature (28 ± 1 °C) for 2 weeks under a natural photoperiod (12 h light and 12 h dark) before being used for this study. During quarantine, the prawns were fed a commercial diet three times per day (at 6:00 a.m., 12:00 p.m., and 6:00 p.m.). The prawns were then randomized into four groups and fed with different diets consisting of *C. asiatica* extract at 0 (commercial diet: CA0), 1 (CA1), 5 (CA5), and 10 (CA10) g/kg feed, 10% of body weight, three times a day. A total of 240 prawns were divided into four groups. Each treatment group consisted of 60 prawns, which were further distributed into three plastic containers (16 × 18 × 30 inches) (20 prawns per tank). The water was partially renewed every two weeks by discarding and replacing 30% of the volume. The water was rigorously disinfected through chlorination followed by dichlorination before use. Water quality was measured before feeding. The pH was 8 ± 0.5, with 5.40 ± 0.78 mg/L dissolved oxygen and a temperature of 29 ± 1 °C. The prawns were studied for a total duration of 28 days. During the rearing period, the gill, hepatopancreas, and hemolymph of each prawn were collected at 12, 24, 48, and 72 h, as well as on days 7, 14, 21, and 28 to analyze gene expression (*n* = 5). All tissues were frozen in liquid nitrogen and stored at −80 °C for further study. On day 28, the prawns were anesthetized on ice, weighed, and hemolymph was collected to study innate immune parameters; percent survival was also recorded. The intestine was dissected and immediately frozen at −80 °C for metagenomic analysis.

The growth performance was assessed using initial body weight (IW) and final body weight (FW) as fundamental parameters. The average weight gain (AWG), average daily gain (ADG), and specific growth rate (SGR) were calculated using the following formulas:

Weight gain (g) = FW − IW

Daily weight gain (g/day) = (FW − IW) × experimental days^−1^

Specific growth rate = 100 × [(ln FW − ln IW) × experimental day^−1^]

The intermolt period (IP) in days was calculated by multiplying the experimental period (EP) by the number of prawns per tank (*n*), then dividing by the total number of molts in that tank (*m*) using the following formula:

IP = (EP × *n*)/*m*

### 2.4. Gene Expression Based on RT-qPCR

#### 2.4.1. RNA Extraction

The RNA in the gill, hepatopancreas, and hemolymph (5 samples/tissue/time × 4 groups) was extracted using Trizol reagent (Invitrogen, Waltham, MA, USA) based on a protocol that was modified from the manufacturer’s instructions. Each sample was homogenized with disposable pellet pestles in 500 μL of Trizol. The solutions were vortexed and left at room temperature for 10 min. Then, 200 μL of chloroform was added, and each mixture was centrifuged at 12,000 rpm for 10 min at 4 °C. The supernatant was transferred into a 1.5 mL tube, followed by the addition of 500 μL of isopropanol. The solution was mixed by vortexing and then stored at −20 °C overnight. After that, the solution was centrifuged at 12,000 rpm for 10 min. The supernatant was discarded, and 500 μL of 75% ethanol was added, then centrifuged at 7500 rpm at 4 °C for 5 min. The supernatant was discarded, and the precipitate was dried at room temperature for 10 min. The RNA precipitate was dissolved in 20 μL of RNase-free water and incubated at 55 °C for 10 min on a heat block. The total RNA was measured for OD absorption at wavelengths of 260 and 280 nm using a NanoDrop 2000 Spectrophotometer (Thermo Scientific™, Waltham, MA, USA). The purity of the RNA was verified by ensuring an A260/A280 ratio of between 1.9 and 2.0, which was used as a criterion for sample selection for cDNA synthesis.

#### 2.4.2. cDNA Synthesis and Gene Expression Analysis

cDNA was generated from the total RNA (500 ng) of each sample using a Tetro cDNA Synthesis Kit (Meridian Bioscience, Cincinnati, OH, USA). The total RNA was denatured at 70 °C for 5 min and immediately cooled on ice. The solution was mixed with 1 µL of oligo (dT) primers, 4 µL of 5× RT Buffer, 1 µL of 10 mM dNTPs, 1 µL of 10 U/µL RNase Inhibitor, 1 µL of 200 U/µL reverse transcriptase, and adjusted to a final volume of 20 µL with nuclease-free water. The reaction was incubated at 45 °C for 30 min, followed by 80 °C for 5 min.

Innate immune-related genes, including *SPI* and *Mr-2α2M*, were analyzed using quantitative real-time PCR, with *ß-actin* serving as the housekeeping gene for normalization (Supplementary Table S2). Real-time PCR reaction was performed in a total volume of 10 µL containing 1 µL of cDNA, 5 µL of 2× SensiFAST SYBR (Meridian Bioscience, Cincinnati, OH, USA), and 1 µL of 10 µM of each forward and reverse primer. The mixture was adjusted to a final volume of 10 µL with nuclease-free water. The primer sequences and expected products are listed in Table 1. The real-time PCR was conducted as follows: pre-denaturation at 95 °C for 5 min, followed by 30 cycles of DNA denaturation at 95 °C for 30 s, primer annealing at 58 and 60 °C for 30 s (Table 1), extension at 72 °C for 30 s, and a final step of melting analysis at 60–95 °C. Analysis of relative gene expression data was calculated by 2^−∆∆Ct^ [21].

### 2.5. Immune Assay from Prawn Hemolymph

#### 2.5.1. Sample Preparation

Hemolymph (250 μL, 5 samples × 4 groups) was collected from the first abdominal segment using a size 22 syringe that had been rinsed with 5% sodium oxalate solution in isotonic saline, serving as a cooled anticoagulant. The collected hemolymph was centrifuged at 10,000 rpm for 10 min at 4 °C to separate the plasma.

#### 2.5.2. Total Protein Analysis

Total protein analysis was conducted according to Bradford (1976), utilizing bovine serum albumin (BSA) concentrations ranging from 200 to 1000 μg/mL as standard solutions [24]. The plasma was diluted 40-fold with 1× phosphate-buffered saline (PBS: 137 mM NaCl, 10 mM phosphate, 2.7 mM KCl at pH 7.4). Subsequently, 20 μL of diluted plasma and standard bovine serum albumin (BSA) solution were each mixed with 200 μL of Bradford reagent in a 96-well plate followed by incubation for 5 min at room temperature. Absorbance readings at 595 nm were then determined using a Take3 plate microplate reader (BioTak, Bristol, UK) with three replicates per sample.

#### 2.5.3. Lysozyme Activity

Lysozyme activity was determined according to Helal and Melzig [25]. A total of 50 μL of the plasma was pipetted into a 96 well-plates containing 200 μL of 0.36 mg/mL *Micrococcus lysodeikticus* prepared with 0.1 M sodium phosphate buffer [25]. The absorbance readings at 530 nm were measured at the initial time (0.5 min) and after 5 min. Reference standards were prepared using egg white lysozyme at concentrations of 2.5, 5, 10, 15, and 20 μg mL^−1^ with 0.1 M sodium phosphate buffer. Lysozyme concentrations (U mL^−1^) were determined from a standard curve, with one unit defined as a 0.001 decrease in absorbance at 530 nm per minute during hydrolysis of *M. lysodeikticus*.

#### 2.5.4. Phenoloxidase (PO) Activity

The residual hemocyte after plasma separation was homogenized using a pestle. The phenoloxidase (PO) activity assay was adapted from Franssens et al. [26]. The hemocyte lysate (50 μL) was combined with 200 μL of 1% trypsin in cacodylate buffer (10 mM sodium cacodylate, 0.45 M sodium chloride, 20 mM calcium chloride, pH 7.0), followed by incubation at room temperature for two minutes to facilitate the lysis of blood cells. Subsequently, 200 μL of L-dihydroxyphenylalanine (L-DOPA) was introduced to generate a dopamine solution, which was then incubated at room temperature for 1 min. OD measurements at a wavelength of 495 nm were conducted at one-minute intervals. For the standard blank, a mixture of 200 μL of cacodylate buffer, 200 μL of trypsin, and 200 μL of L-DOPA was prepared and incubated at room temperature for one minute, with results calculated in units per minute (U min ^−1^ mg ^−1^ protein) [26].

### 2.6. Metagenomic Analysis of Gut Microbiota Based on 16S rRNA Sequencing

Metagenomic analysis was conducted to compare the intestine bacterial composition between the CA0 and CA10 groups. Genomic DNA was isolated from prawn intestine (5 samples/groups) using the QIAamp DNA stool Mini Kit (Qiagen, Hilden, Germany). DNA quality was estimated using a NanoDrop 2000 Spectrophotometer (Thermo Scientific™, USA) at wavelengths of 260 and 280 nm. The V3-V4 fragment of bacterial 16S rRNA was amplified. For preprocessing of reads for fragment analyses, we used a DADA2 V 1.20.0 pipeline. The fragments were checked for quality trimming and filtering techniques using 250 bp for forward reads and 240 bp for reverse reads. Paired reads were merged to consolidate sequences into full complete entities. The fragments were then subjected to the chimera removal process by comparing each sequence with higher abundance counterparts to identify and eliminate potential artifacts. Analysis of sequencing data was performed using QIIME, and bar charts, pie charts, and variable-radius pie charts were generated using R software [27]. Heat tree and meta-diversity plots were created using the R packages ape [28] and metacoder [29] and adapted from the method published by Ugyen et al. [30], which displays the abundance of organisms.

### 2.7. Statistical Analysis

Growth and immune parameters were analyzed using one-way analysis of variance (ANOVA) followed by Tukey’s honestly significant difference (HSD) post hoc test to identify significant differences between groups. For the relative gene expressions, a one-way ANOVA was performed for each time point to determine the effects of the different dietary treatments. Significant differences among groups at each specific time point were identified using a Tukey’s HSD post hoc test. All statistical analyses were performed using R (version 3.0.2) and modified using additional packages in R [27]. Throughout the experiment, a *p*-value of less than 0.05 was considered statistically significant.

## 3. Results

### 3.1. Effect of C. asiatica Crude Extract Supplementation on Growth Performance of Giant Freshwater Prawns

We found that the initial weight of prawns ranged from 2.71 to 2.98 g. After a 28-day feeding period, prawns fed with *Centella asiatica* extract had an average weight ranging from 4.06 to 4.35 g. The HSD results showed no significant differences in ADG, survival rate, or IP between the experimental and control groups, as all *p*-values were greater than 0.05 (*p* > 0.05). However, SGR showed significant variation, with the CA10 group exhibiting the highest value, which was significantly different from the other groups (*p* < 0.05) (Table 2).

### 3.2. Mr-2α2M and SPI Gene Expression Levels in the Tissues over a Feeding Period of 12 h to 28 Days

**Figure 1:**
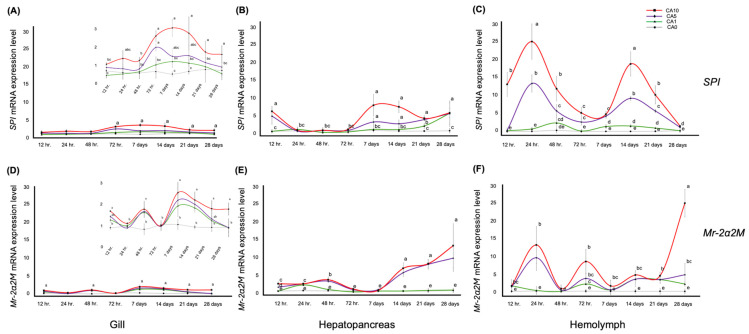
Gene expression analysis results for various prawn tissues including gill (**A**,**D**), hepatopancreas (**B**,**E**), and hemolymph (**C**,**F**) for difference feeding times (*x*-axis). The expression levels of the targeted genes, *SPI* (**A**–**C**), and *Mr-2α2M* (**D**–**F**) were quantified using SYBR-based RT-qPCR, with *actin* as the housekeeping gene. Relative levels of mRNA expression (*y*-axis) were normalized by the CA0 group at the 12 h feeding time. Significant differences (*p* < 0.05) in gene expression level are indicated by different letters.

The results revealed statistically significant differences depending on the feeding time and concentration of *C. asiatica* (*p* < 0.05) across all tissues. In gill tissue, *SPI* gene expression remained low in all groups for the first 48 h. CA5 and CA10 showed a small increase at 72 h, with CA10 reaching the highest level on day 7. The CA5 group showed a similar pattern to CA10, but with a lower peak. In contrast, the CA0 and CA1 groups displayed consistently low expression levels throughout the feeding period (Figure 1A). In the hepatopancreas, CA5 and CA10 exhibited an initial peak at 12 h, followed by a higher peak on day 7. The CA0 and CA1 groups maintained consistently low and stable expression levels until day 21; only CA1 increased, showing a higher peak after day 28 (Figure 1B). In hemolymph, the *SPI* expression under the CA10 treatment showed peaks at 12 h, with the highest peak at 24 h. Afterward, the gene expression decreased and then peaked again on day 14 before declining. CA5 exhibited a similar trend to CA10, but with lower expression levels. In contrast, the CA1 and CA0 groups showed consistently low expression levels throughout the study period (Figure 1C). The *Mr-2α2M* gene expression showed fluctuating peaks across all groups, with trends of rising and falling expression in both gill and hemolymph. However, hepatopancreas showed initially low and stable expression levels, which gradually increased over time. For gill and hemolymph, the CA10 group exhibited a higher peak, while the CA1 and CA5 groups peaked simultaneously, but at lower levels. The highest peak was observed on day 7 in the gills and on day 28 in the hemolymph. Interestingly, a marked reduction in expression levels was observed in the CA1 and CA5 groups 28 days post-feeding, indicating a downward trend compared to previous time points (Figure 1D,F). In contrast, the CA10 group maintained high expression levels in both gill and hemolymph. The CA0 treatment consistently exhibited low expression compared to the treatment groups. In the hepatopancreas, the CA5 and CA10 groups showed a significant peak in *Mr-2α2M* expression after 14 days of feeding compared to CA0 and CA1, reaching the highest level on day 28. The CA5 and CA10 groups showed similar expression levels on day 21, while the CA0 and CA1 groups exhibited low and stable expression levels throughout the feeding period (Figure 1E). In Table 3, statistical comparisons were performed among the dietary treatments at the same sampling time for the same gene and organ. This analysis assessed whether the concentration of *C. asiatica* in the diet influenced mRNA expression levels under identical temporal and tissue conditions. Overall, CA10 frequently exhibited expression levels that were significantly different from the other concentrations, with a consistent trend toward higher expression values, suggesting that the highest dietary concentration tended to stimulate gene expression more effectively than the lower concentrations.

### 3.3. Innate Immune Parameters Assay

**Figure 2:**
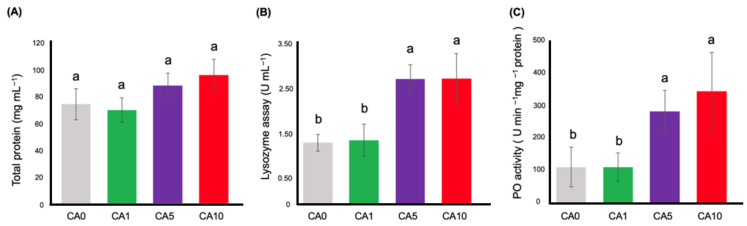
Innate immune parameters: total protein analysis (**A**), lysozyme activity (**B**) and phenoloxidase activity (**C**) of *M. rosenbergii* fed with added *C. asiatica* at 28 days after feeding. Values represent means (±S.D.) for four diets (CA0, CA1, CA5, and CA10). Statistically significant differences (*p* < 0.05) between the treatment groups and control group are indicated by different letters.

The impact of *C. asiatica* supplementation on the immune parameters of prawns was assessed using biochemical assays after 28 days of feeding (Supplementary Table S3). The total protein levels were not significantly different (*p* > 0.05) compared to CA0 (Figure 2A). However, the results showed that the crude extract of *C. asiatica* led to a statistically significant increase in lysozyme activity (*p* < 0.05) and PO activity (*p* < 0.05) (Figure 2B,C). Lysozyme and PO activities in the CA5 and CA10 groups were higher than in the CA0 group.

### 3.4. Intestine Microbiome Analysis Based on 16S rRNA Gene Sequencing

**Figure 3:**
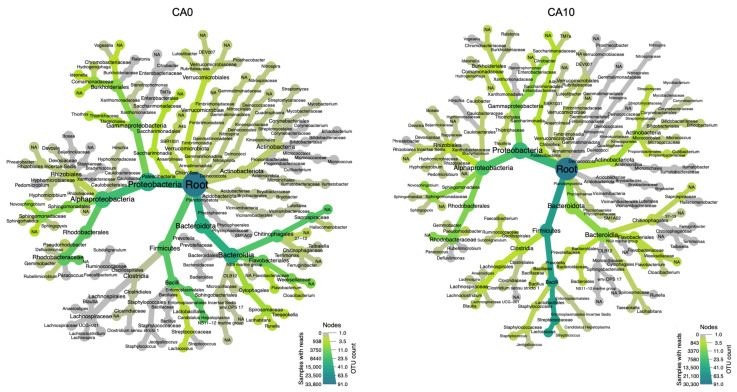
Comparison of the taxonomic composition of intestine microbiota in *M. rosenbergii* between CA0 and CA10 groups. A heat tree illustrates the metagenomic data, where the size and color of nodes and edges represent the relative abundance of organisms in each treatment group.

**Figure 4:**
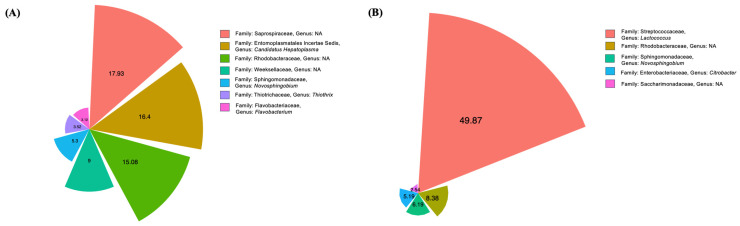
Variable-radius pie charts comparing the abundance of bacterial genera between the CA0 (**A**) and CA10 groups (**B**).

As depicted in Supplementary Figure S1, high-throughput sequencing was employed for both the CA0 and CA10 groups. Through comprehensive analysis, samples yielded 75,865 clean data points. Bacterial diversity analysis revealed the presence of 21 phyla in the intestine samples, with the top 50 taxa showing distinct patterns (Supplementary Figure S2). The heat tree analysis showed the bacterial compositions with relative abundance of at least > 0.2%. In this representation, the nodes correspond to different taxonomic levels, and the branches illustrate the relationships between them. The abundance of operational taxonomic units (OTUs) is depicted through the size, color, and intensity of the nodes, starting from the phylum level (Figure 3). The cleaned data were subsequently displayed to show the abundance at the class (Supplementary Figure S3), order (Supplementary Figure S4), and family levels (Supplementary Figure S5). In the CA0 group, the genus Candidatus Hepatoplasma was the dominant within the Firmicutes phylum, while members of the Proteobacteria phylum also exhibited high relative abundance. Within the Bacteroidota phylum, the bacterial community was largely composed of the Saprospiraceae family, although no dominant genus was specifically identified. The CA10 group had significant abundances of Firmicutes and Proteobacteria, comprising more than half of the bacterial community (Figure 3). At the taxonomic levels ranging from family to genus, significant shifts in microbial composition were observed. The CA0 group had the highest abundance of the Saprospiraceae family (17.93%). Interestingly, disadvantageous bacteria such as Candidatus Hepatoplasma (16.4%), Weeksellaceae (9%), Thiothrix (3.52%), and Flavobacterium (3.12%) were also found in high numbers. A notable decline in the population of the Saprospiraceae family and Candidatus Hepatoplasma were observed, accompanied by an increase in the abundance of Lactococcus in the CA10 group. Although Lactococcus was initially present at low levels in the CA0 group, it became the dominant bacterial genus in CA10, reaching 49.87% (Figure 4). The taxonomic composition and abundance of the selected bacterial genera under the CA0 and CA10 treatments are compared in Supplementary Table S4.

## 4. Discussion

*C. asiatica* has been used as a supplement in food for animals such as chicken [31] and piglets [32]. A literature review showed the effectiveness of dietary supplementation with *C. asiatica* in enhancing the immune system of aquatic species including white leg shrimp [16] and Nile tilapia [15]. In this study, *C. asiatica* crude extract was used as a dietary component for giant freshwater prawns to evaluate growth performance and immune parameters through gene expression and enzyme activity tests. The triterpenoid bioactive compounds present in *C. asiatica* are asiatic acid, madecassic acid, asiaticoside, and madecassoside [33], which eliminate excess ROS and prevent oxidative stress like other phytotherapy plants [34]. However, higher concentrations of crude extract supplements in food can adversely impact the feeding of animals due to the presence of saponins, which reduce food palatability in crustaceans [35]. The results showed that there were no statistically significant variations in IP or survival rate, indicating that prawns grew normally when fed supplemental food containing *C. asiatica*. The absence of any negative effects on the survival rate indicates that the supplementary food did not negatively impact the prawns’ health. Our results showed no statistically significant variations in ADG, but there was a statistically significant increase in SGR, indicating that supplementation with *C. asiatica* positively influenced prawn growth performance. The efficacy of herbal extracts as dietary supplements in enhancing growth largely depends on the type of herb and the extraction method employed. Polar-extracted herbal diets have been shown to significantly improve ADG and survival rates in *Penaeus monodon* when compared to those extracted with non-polar chemicals [36]. These feed additives provide a source of nutrients and beneficial phytochemicals, promoting growth performance and immune system functioning of animals in aquaculture. Various plants such as Bermuda grass (*Cyanodon dactylon*) [37], fenugreek (*Trigonella foenum-graecum*), garlic (*Allium sativum*), turmeric (*Curcuma longa*), ginger (*Zingiber officinale*) [38], and black catnip (*Phyllanthus amurus*) [39] have been supplemented in the diet to improve health and growth in giant freshwater prawns [37,38,39].

Our studies also measured three well-known immune parameters based on total protein analysis, lysozyme assay, and PO activity measurements in prawns that received *C. asiatica* extract. Dietary supplementation with *C. asiatica* at all concentrations did not change the total protein content. This result indicated that the prawns in the experiments grew without stress. Hemolymph protein levels are generally sensitive to environmental factors like molting, infection, hypoxia, and salinity variations [40,41]. When considering the lysozyme assay and PO activity, the results revealed increased non-specific defense activity when the prawns received sufficient supplementation of *C. asiatica* in the CA5 and CA10 groups. This finding is similar to previous research on Nile tilapia, which found that adding 10 g/kg *C. asiatica* powder in the feed significantly improved serum lysozyme levels [15]. This finding aligns with a previous study on β-glucans, which demonstrated enhanced bactericidal activity in black tiger shrimp lasting up to 18 days following immersion. These results suggest that dietary supplementation requires time to elicit measurable positive effects [42]. High levels of the phenol oxidase enzyme were found in the CA5 and CA10 groups. The phenol oxidase enzyme forms through the PO activating system process, which can be stimulated by microbial components such as lipopolysaccharides (LPS) or peptidoglycans and B-1,3-glucans [42]. The PO activating system process also stimulates cells that destroy and ingest foreign substances [43]. It has been observed that carbohydrate compounds can stimulate the activity of the phenol oxidase enzyme [44]. *C. asiatica* contains numerous chemicals, with saponin glycosides, asiaticoside, and madecassoside being important. This may impact phenol oxidase enzyme levels. Consequently, research revealed higher levels of phenol oxidase enzymes and lysozyme activity in the experimental groups compared to the control group after adding food supplements in *L. vannamei* [45,46] and *Penaeus vannamei* [47].

During 28 days of feeding, the innate immune-related gene expression levels in the gill, hepatopancreas, and hemolymph tissues of prawns were examined following stimulation with a food formula containing crude *C. asiatica* extract powder. The results showed that the 5 and 10 g/kg feed had enhanced expressions of the *SPI* and *Mr-2α2M* genes in all organs. The gene expression levels were significantly higher in the hemolymph and hepatopancreas compared to the gill tissue, and they exhibited more pronounced fluctuations. This result suggests a tissue-specific regulatory mechanism, with the hepatopancreas and hemolymph more actively involved in the physiological or immune response. *SPI* and *Mr-2α2M* expression indicated a possible dose-dependent response. The temporal peaks observed in the expression levels suggested a dynamic regulatory process that may be critical for organism adaptation and response. This supported the idea that gene expression differs in response patterns such as long-term upregulation, short-term up- or downregulation, or no response. Response times can range from less than a day to up to 7 days, and these patterns target specific tissues, as assessed by mRNA expression [48]. This observation concurred with previous studies on gene expression patterns in the hepatopancreas of river prawns (*Macrobrachium nipponense*) under nitrite stress, highlighting the organ’s crucial role in detoxification and immunity [49]. In addition, our findings aligned with the reported immune-stimulating properties of *C. asiatica*. The main active compounds in this herb, including polysaccharides and terpenoids such as madecassoside, stimulated both innate and specific immunity [50]. The *Mr-2α2M* gene encodes proteins that are crucial for inhibiting various protease enzymes, thereby playing a significant role in the innate immune system’s defense mechanisms [22]. This gene is involved in mediating essential immune functions such as T-cell proliferation and enhancing the activity of white blood cells and macrophages [51]. Studies on crabs (*Scylla paramamosain*) infected with *Vibrio parahaemolyticus* showed a notable upregulation of the immune-related *proPO* and *Mr-2α2M* genes 72 h post-infection [52]. Also, *SPIs* are key components of the shrimp immune system, playing an essential role in defending against pathogens. They inhibit bacterial growth by binding to bacteria and blocking microbial serine proteases necessary for bacterial survival [53]. Additionally, *SPIs* act as immune modulators by regulating the proPO system, which is a critical component of the innate immune response in crustaceans [54].

This study utilized high-throughput sequencing to investigate the impact of *C. asiatica* supplementation on the intestine bacterial community of prawns in the CA0 and CA10 groups at the end of feeding. The analysis uncovered the presence of numerous phyla bacteria, presenting a diverse microbial community within the prawn’s intestine. After supplementation with *C. asiatica*, the populations of Saprospiraceae and *Candidatus Hepatoplasma* declined, with an increased abundance of *L. lactis*. The results showed potential benefits of plant extracts for improved intestine health by increasing beneficial bacteria. *Candidatus Hepatoplasma* signifies low-nutrient conditions within the host [55], and it is a marker of irregularities in nutrient absorption [56]. The significant decrease in *Candidatus Hepatoplasma* was also accompanied by a reduction in several disadvantageous bacteria, including Flavobacteria, which cause columnaris disease [57], and *Thiothrix* sp., which are associated with inflammation and melanization in bacterial gill disease [58]. In contrast, beneficial bacteria including *Lactococcus* sp. increased after feeding with *C. asiatica*. This Gram-positive bacterium is known for its probiotic potential and use in food fermentation and can enhance health and improve disease resistance when used as a dietary supplement [59,60]. These findings suggest that *C. asiatica* supplementation may exert selective pressure on the intestinal microbiota ecosystem, leading to increased prawn health and performance. Further research is required to elucidate the mechanisms underlying these changes and assess the functional consequences for prawn physiology and metabolism. This study contributes to our understanding of the complex interactions between dietary supplements and the intestinal microbiota in aquatic organisms, with potential implications for aquaculture practices and prawn health management.

## 5. Conclusions

This study demonstrated that dietary supplementation with a crude extract of *C. asiatica* has beneficial effects on the health and performance of *M. rosenbergii*. Although no significant differences were observed in average daily gain, survival rate, or intestinal permeability, prawns fed with the highest supplementation level (10 g kg^−1^) showed a significantly improved specific growth rate. Furthermore, immune parameters such as lysozyme and phenoloxidase activities and the expression of immune-related genes were significantly enhanced in the groups receiving 5 and 10 g kg^−1^ of the extract. In addition, gut microbiota analysis revealed an increase in beneficial bacteria such as *Lactococcus* sp., accompanied by a reduction in potentially harmful taxa including Candidatus Hepatoplasma, Flavobacteriaceae, Weeksellaceae, and *Thiothrix* sp. These findings suggest the potential of *C. asiatica* crude extract as an immunostimulatory agent and as a modulator of microbial communities in aquaculture systems. The enhancement of immune function and a more balanced gut microbiota ultimately contribute to healthier and more resilient prawns. Our study, therefore, highlights the promising preventive roles of *C. asiatica* crude extract in aquaculture practices. It emphasizes its potential to enhance the health and productivity of aquatic organisms and its application in aquaculture management strategies, particularly by strengthening the animal’s natural defenses against disease.

## Figures and Tables

**Table 1:** Primer sequences used for gene expression measurement based on real-time quantitative PCR assays.

Gene Name	Primer Name	Primer Sequences 5′–3′	Annealing Temperature (°C)	Melting Temperature (°C)	Accession Number	Reference
*Mr-2α2M*	*Mr-2α2M* forward	GATATGAAGTTGATGGAAA	60	78.4	ABK60046.1	Likittrakulwong et al., 2017 [22]
	*Mr-2α2M* reverse	GTGAACTCTGGCTGGTAGTAA				
*SPI*	*SPI* forward	CACTTTAAGCCCGTGTGCGGTAAT	58	84.5	FJ429307.1	Tewpair et al., 2008 [23]
	*SPI* reverse	TGAACATCTTCGAGTGGGAACACC				
*ß-actin*	*ß-actin* forward	TTCACCATCGGCATTGAGAGGTTC	60	85.1	KY038927.1	Likittrakulwong et al., 2017 [22]
	*ß-actin* reverse	CACGTCGCACTTCATGATGGAGTT				

**Table 2:** Growth performance and survival rate indices of prawns fed diets containing *C. asiatica* extract for 28 days.

Parameter	Groups			
	CA0	CA1	CA5	CA10
Initial body weight (g)	2.84 ± 0.47	2.71 ± 0.45	2.98 ± 0.52	2.85 ± 0.34
Final body weight (g)	4.01 ± 0.58	4.06 ± 0.75	4.35 ± 0.83	4.32 ± 0.81
Average weight gain (g)	1.17 ± 0.142	1.35 ± 0.124	1.38 ± 0.131	1.47 ± 0.192
Average daily gain (g day^−1^)	0.041 ± 0.010	0.048 ± 0.010	0.049 ± 0.013	0.052 ± 0.01
Specific growth rate (% day^−1^)	1.23 ± 0.02 ^c^	1.41 ± 0.02 ^b^	1.42 ± 0.01 ^b^	1.48 ± 0.02 ^a^
Survival rate (%)	64.44 ± 5.09	66.67 ± 3.33	64.44 ± 5.09	65.55 ± 1.92
Intermolt period (Days)	7.33 ± 0.57	6.66 ± 0.57	7.33 ± 0.57	7.66 ± 0.57

Significant differences (*p* < 0.05) in each growth parameter are indicated by different letters.

**Table 3:** Statistical comparison of relative mRNA expression of *SPI* and *Mr-2α2M* genes among dietary *C. asiatica* treatments across different organs and sampling times.

Feeding Day	Gene	Organ	CA0	CA1	CA5	CA10
12 h.	*SPI*	Gill	1.00 ± 0.06 ^b^	0.76 ± 0.14 ^ab^	1.18 ± 0.27 ^ab^	1.35 ± 0.05 ^ab^
		Hepatopancreas	1.00 ± 0.47 ^b^	1.03 ± 0.46 ^b^	4.98 ± 2.13 ^a^	6.25 ± 1.30 ^a^
		Hemolymph	1.00 ± 0.37 ^b^	1.20 ± 0.215 ^b^	1.30 ± 0.62 ^b^	13.51 ± 3.15 ^a^
	*Mr-2α2M*	Gill	1.00 ± 0.11 ^c^	1.42 ± 0.13 ^b^	1.60 ± 0.06 ^b^	1.88 ± 0.04 ^a^
		Hepatopancreas	1.00 ± 0.05 ^c^	0.99 ± 0.06 ^c^	2.05 ± 0.16 ^b^	3.00 ± 0.53 ^a^
		Hemolymph	1.00 ± 0.28 ^b^	2.50 ± 0.83 ^a^	2.25 ± 0.87 ^a^	2.53 ± 1.66 ^a^
24 h.	*SPI*	Gill	0.91 ± 0.28 ^a^	0.84 ± 0.29 ^a^	1.11 ± 0.22 ^a^	1.65 ± 1.23 ^a^
		Hepatopancreas	1.13 ± 0.26 ^a^	1.50 ± 0.67 ^a^	1.07 ± 0.24 ^a^	1.31 ± 0.41 ^a^
		Hemolymph	1.03 ± 0.2 ^c^	1.66 ± 0.48 ^c^	13.74 ± 2.32 ^b^	24.88 ± 4.7 ^a^
	*Mr-2α2M*	Gill	1.06 ± 0.20 ^a^	1.12 ± 0.05 ^a^	0.99 ± 0.04 ^a^	1.23 ± 0.05 ^a^
		Hepatopancreas	1.11 ± 0.22 ^b^	2.68 ± 0.26 ^a^	2.98 ± 0.14 ^a^	2.99 ± 0.33 ^a^
		Hemolymph	1.11 ± 0.31 ^b^	1.27 ± 1.06 ^b^	10.03 ± 3.40 ^a^	13.47 ± 4.95 ^a^
48 h.	*SPI*	Gill	0.89 ± 0.04 ^b^	0.96 ± 0.38 ^b^	1.11 ± 0.16 ^b^	1.55 ± 0.17 ^a^
		Hepatopancreas	1.15 ± 0.27 ^a^	0.75 ± 0.34 ^a^	1.25 ± 0.72 ^a^	1.30 ± 0.41 ^a^
		Hemolymph	1.26 ± 0.59 ^c^	3.26 ± 1.99 ^bc^	6.44 ± 1.72 ^b^	12.36 ± 3.3 ^a^
	*Mr-2α2M*	Gill	0.92 ± 0.30 ^b^	1.81 ± 0.26 ^a^	1.86 ± 0.24 ^a^	1.98 ± 0.43 ^a^
		Hepatopancreas	1.04 ± 0.15 ^b^	1.42 ± 0.34 ^b^	3.71 ± 0.25 ^a^	4.10 ± 0.76 ^a^
		Hemolymph	1.02 ± 0.32 ^a^	1.05 ± 0.36 ^a^	1.35 ± 0.38 ^a^	1.75 ± 1.04 ^a^
72 h.	*SPI*	Gill	0.96 ± 0.34 ^b^	1.32 ± 0.44 ^b^	2.24 ± 0.19 ^a^	2.84 ± 0.18 ^a^
		Hepatopancreas	1.16 ± 0.33 ^a^	0.87 ± 0.39 ^a^	1.26 ± 0.30 ^a^	1.55 ± 0.12 ^a^
		Hemolymph	1.01 ± 0.18 ^b^	1.12 ± 0.42 ^b^	3.49 ± 0.81 ^a^	5.93 ± 1.42 ^a^
	*Mr-2α2M*	Gill	1.15 ± 0.26 ^a^	1.09 ± 0.10 ^a^	1.08 ± 0.23 ^a^	1.11 ± 0.13 ^a^
		Hepatopancreas	1.04 ± 0.13 ^a^	0.77 ± 0.10 ^a^	1.39 ± 0.30 ^a^	1.55 ± 0.21 ^a^
		Hemolymph	1.08 ± 0.46 ^c^	2.99 ± 0.24 ^b^	4.47 ± 1.37 ^ab^	9.02 ± 3.24 ^a^
7 days	*SPI*	Gill	0.83 ± 0.11 ^c^	1.49 ± 0.29 ^b^	1.77 ± 0.41 ^b^	3.27 ± 0.43 ^a^
		Hepatopancreas	1.25 ± 0.42 ^b^	1.51 ± 0.68 ^b^	3.44 ± 1.06 ^a^	7.83 ± 2.82 ^a^
		Hemolymph	1.01 ± 0.16 ^c^	2.30 ± 0.52 ^b^	5.07 ± 0.6 ^a^	5.56 ± 0.83 ^a^
	*Mr-2α2M*	Gill	1.17 ± 0.28 ^b^	2.16 ± 0.58 ^a^	2.46 ± 0.32 ^a^	2.86 ± 0.56 ^a^
		Hepatopancreas	1.02 ± 0.10 ^a^	0.98 ± 0.20 ^a^	1.37 ± 0.21 ^a^	1.17 ± 0.05 ^a^
		Hemolymph	1.02 ± 0.51 ^b^	1.42 ± 1.34 ^a^	1.43 ± 0.56 ^a^	2.47 ± 1.56 ^a^
4 days	*SPI*	Gill	0.97 ± 0.14 ^c^	1.41 ± 0.22 ^bc^	1.80 ± 0.55 ^ab^	2.99 ± 0.88 ^a^
		Hepatopancreas	1.09 ± 0.23 ^b^	1.49 ± 0.66 ^b^	2.98 ± 1.01 ^a^	7.43 ± 1.55 ^a^
		Hemolymph	1.01 ± 0.17 ^d^	2.45 ± 0.41 ^c^	9.79 ± 0.58 ^b^	18.92 ± 3.23 ^a^
	*Mr-2α2M*	Gill	1.04 ± 0.11 ^b^	2.05 ± 0.23 ^a^	2.24 ± 0.16 ^a^	2.47 ± 0.44 ^a^
		Hepatopancreas	1.03 ± 0.22 ^b^	0.99 ± 0.11 ^b^	6.02 ± 1.01 ^a^	7.22 ± 1.56 ^a^
		Hemolymph	1.03 ± 0.53 ^b^	4.28 ± 1.98 ^a^	4.29 ± 0.97 ^a^	5.41 ± 1.22 ^a^
21 days	*SPI*	Gill	1.07 ± 0.66 ^b^	1.22 ± 0.29 ^b^	1.44 ± 0.22 ^b^	2.00 ± 0.57 ^a^
		Hepatopancreas	1.08 ± 0.22 ^c^	2.44 ± 0.67 ^b^	4.08 ± 0.23 ^a^	4.42 ± 0.66 ^a^
		Hemolymph	1.03 ± 0.22 ^d^	1.89 ± 0.11 ^c^	6.42 ± 0.44 ^b^	10.70 ± 2.44 ^a^
	*Mr-2α2M*	Gill	1.02 ± 0.23 ^b^	1.38 ± 0.55 ^ab^	1.47 ± 0.45 ^ab^	2.01 ± 0.43 ^a^
		Hepatopancreas	1.02 ± 0.44 ^b^	1.17 ± 0.55 ^b^	8.33 ± 1.32 ^a^	8.52 ± 1.05 ^a^
		Hemolymph	1.03 ± 0.82 ^b^	4.15 ± 1.01 ^a^	4.20 ± 0.76 ^a^	5.22 ± 1.01 ^a^
28 days	*SPI*	Gill	1.02 ± 0.26 ^b^	0.85± 0.32 ^b^	1.2 ± 0.31 ^b^	1.87± 0.46 ^a^
		Hepatopancreas	1.16 ± 0.6 ^b^	5.71 ± 2.78 ^a^	5.65 ± 3.31 ^a^	5.84 ± 1.23 ^a^
		Hemolymph	1.19 ± 0.45 ^b^	1.20 ± 0.18 ^b^	2.09 ± 0.2 ^a^	2.41± 0.11 ^a^
	*Mr-2α2M*	Gill	1.02 ± 0.26 ^b^	1.00 ± 0.18 ^b^	1.00 ± 0.48 ^b^	1.99 ± 0.33 ^a^
		Hepatopancreas	1.16 ± 0.6 ^b^	1.26 ± 0.37 ^b^	10.02 ± 3.59 ^a^	13.46 ± 6.06 ^a^
		Hemolymph	1.01 ± 0.66 ^c^	2.99 ± 0.53 ^b^	5.47 ± 3.06 ^b^	24.711 ± 3.71 ^a^

Statistically significant differences (*p* < 0.05) among dietary treatments for the same organ, gene, and sampling time are indicated by different superscript letters.

## Data Availability

The data presented in this study are available upon request from the corresponding author.

## Supplementary Materials

The following supporting information can be downloaded at: https://www.mdpi.com/article/10.3390/ani15172507/s1. Figure S1: Data quality control; Figure S2: Top 50 amplicon sequence variant heatmap of 16S rRNA gene reads assigned to taxonomy on the bacterial amplicon sequence variant number; Figure S3: Bacterial characterization in the intestine of prawns was conducted using two groups: one fed with 10 g/kg supplementation food, and the other fed non-supplemented food as a control. The proportions of bacterial taxa at the class level were analyzed in each sample (*n* = 5). Bar chart inserts are used to illustrate the proportions of bacterial genera present with a relative abundance of at least > 0.2%; Figure S4: Bacterial characterization in the intestine of prawns was conducted using two groups: one fed with 10 g/kg supplementation food, and the other fed non-supplemented food as a control. The proportions of bacterial taxa at the order level were analyzed in each sample (*n* = 5). Bar chart inserts were used to illustrate the proportions of bacterial genera present with a relative abundance of at least > 0.2%; Figure S5: Bacterial characterization in the intestine of prawns was conducted using two groups: one fed with 10 g/kg supplementation food, and the other fed non-supplemented food as a control. The proportions of bacterial taxa at the family level were analyzed in each sample (*n* = 5). Bar chart inserts are used to illustrate the proportions of bacterial genera present with a relative abundance of at least > 0.2%; Table S1: Centelloside content in *Centella asiatica* leaves extracted using ethanol; Table S2: Primer sequences used for gene expression measurement in real-time quantitative PCR assays; Table S3: The immune indexes assay; Table S4: Taxonomic composition and abundance of selected bacterial genera under CA0 and CA10 treatments.

## Abbreviations

The following abbreviations are used in this manuscript:

IW	Initial body weight
FW	Final body weight
AWG	Average weight gain
ADG	Average daily gain
SGR	Specific growth rate
IP	Intermolt period
EP	Experimental period
*n*	Number of prawns per tank
*m*	Total number of molts per tank
pro-PO	Prophenoloxidase
