# Supplementary material for: Dietary Supplementation with Gotu Kola (Centella asiatica) Extract Enhanced Innate Immune Responses, Modulated Immune-Related Gene Expression, and Improved Gut Microbiota in Giant Freshwater Prawn (Macrobrachium rosenbergii)
Source: Animals (Basel). 2025 Aug 26;15(17):2507. doi: 10.3390/ani15172507 (PMC12427494; doi:10.3390/ani15172507)
Supplement: Supplementary file 1 [file animals-15-02507-s001.zip › animals-3776522-supplementary.pdf]

Supplementary

Dietary Supplementation with Gotu Kola (Centella asiatica) Extract Enhanced Innate Immune Responses, Modulated Immune-Related Gene Expression, and Improved Gut Microbiota in Giant Freshwater Prawn (Macrobrachium rosenbergii)

Table S1. Centelloside content on *Centella asiatica* leaves extracted by ethanol.

Sample	rep	DW	Composition				
			Peak height				
			Madecassoside	Madecassic acid	Asiatic acid	Asiaticoside	
C. asiatica	1	0.1	229.000	33.500	34.200	201.500	
C. asiatica	2	0.1	163.600	45.800	45.500	172.200	
C. asiatica	3	0.1	134.300	66.700	61.200	147.300	
Sample			%DW (g/100g)				
			Madecassoside	Madecassic acid	Asiatic acid	Asiaticoside	Centelloside
C. asiatica	1	0.1	1.2196	0.1214	0.1474	1.0285	2.5169
C. asiatica	2	0.1	0.8713	0.1659	0.1962	0.8789	2.1123
C. asiatica	3	0.1	0.7152	0.2416	0.2638	0.7518	1.9726

Table S2. Primer sequences used for gene expression measurement by real-time quantitative PCR assays

Gene name	Primer name	Primer sequences 5'- 3'	Annealing temperature	Melting temperature (Tm)
Mr-2α2M	*Mr-2α2M* forward	GATATGAAGTTGATGGAAA	60	78.4
	Mr-2α2M reverse	GTGAACTCTGGCTGGTAGTAA		
SPI	*SPI* Forward	CACTTTAAGCCCGTGTGCGGTAAT	58	84.5
	SPI Reverse	TGAACATCTTCGAGTGGGAACACC		
ß-Actin	*ß-Actin* forward	TTCACCATCGGCATTGAGAGGTTC	58-60	85.1
	ß-Actin Reverse	CACGTCGCACTTCATGATGGAGTT		

Table S3. The immune indexes assay

Treatment	Immune indexes		
	Total protein (mg mL^{-1})	lysozyme assay (U mL^{-1})	Phenoloxidase activity (U min^{-1} mg^{-1} protein)
CA0	73.73 ± 11.57	1.35 ± 0.18	110.33 ± 60.11
CA1	69.41 ± 8.92	1.40 ± 0.34	110.16 ± 42.71
CA5	87.43 ±9.27	2.76 ± 0.30*	280.00 ± 63.61*
CA10	95.41 ±11.69	2.76 ± 0.55*	340.26 ± 117.19*

* Represents a statistically significant difference analysis by the Mann-Whitney U Test

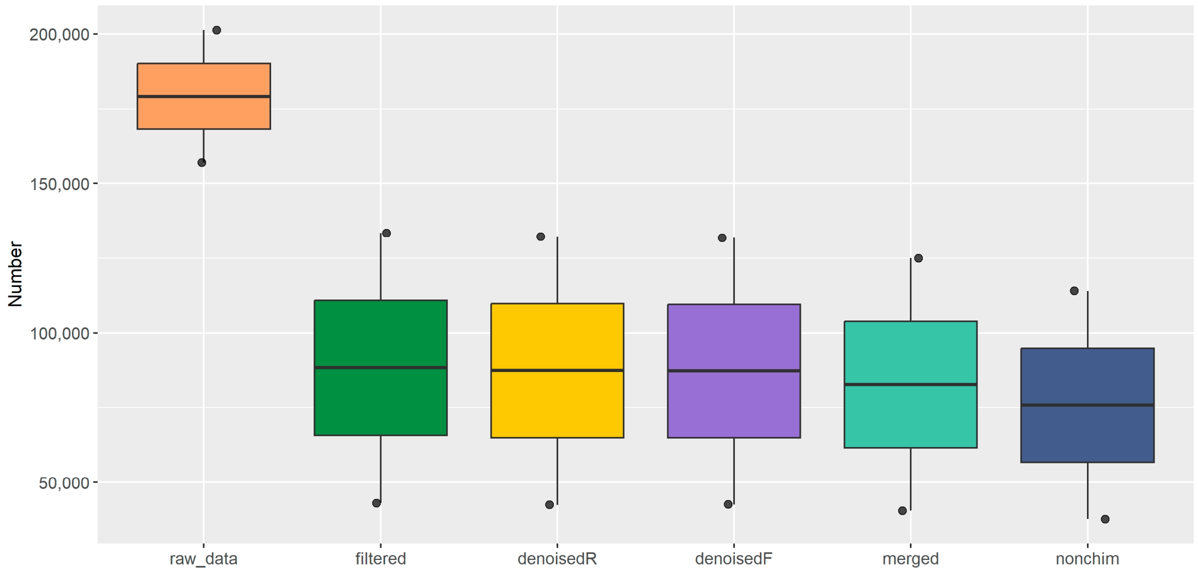

Figure S1 Data quality control

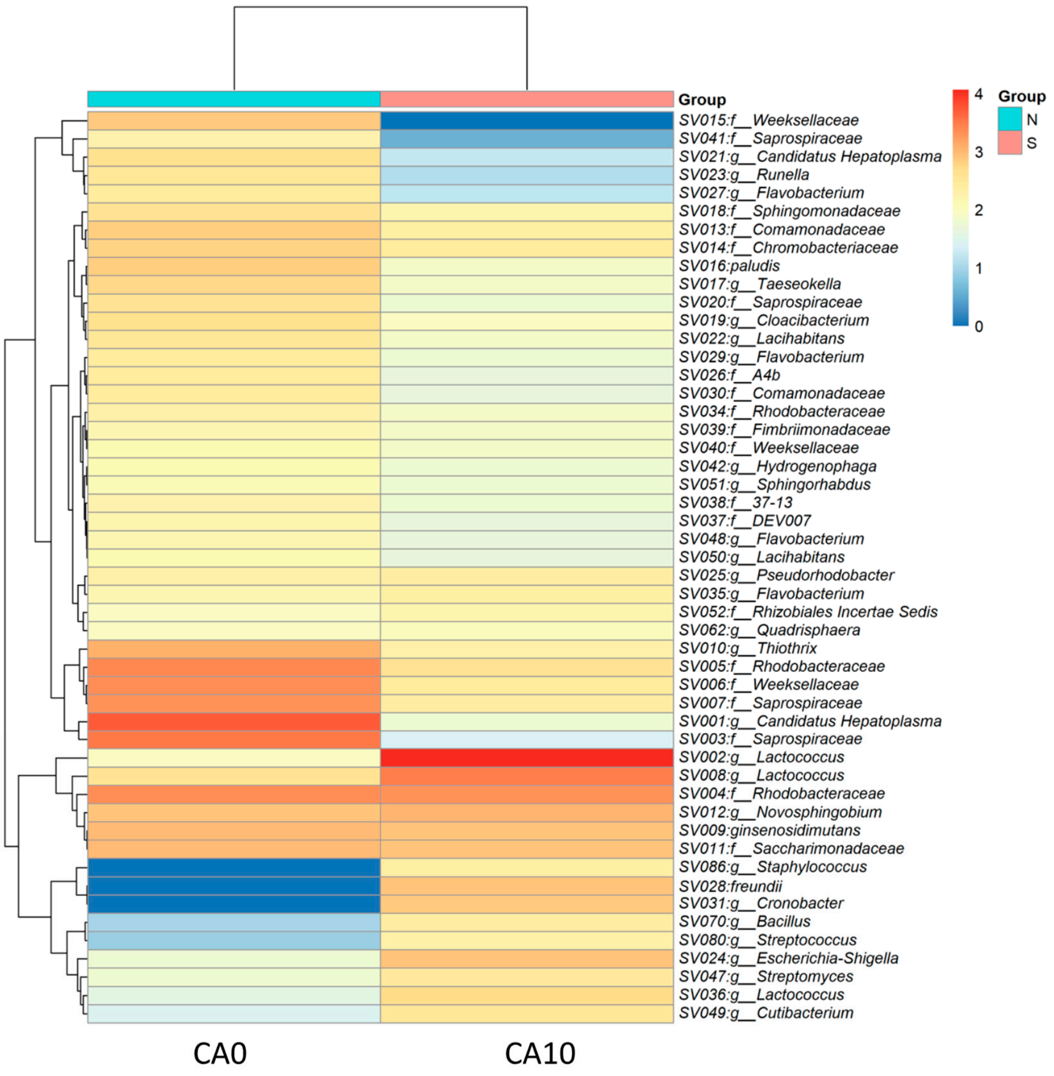

Figure S2 Top 50 amplicon sequence variant Heatmap of 16S rRNA gene reads assigned to taxonomy on the bacterial amplicon sequence variant number.

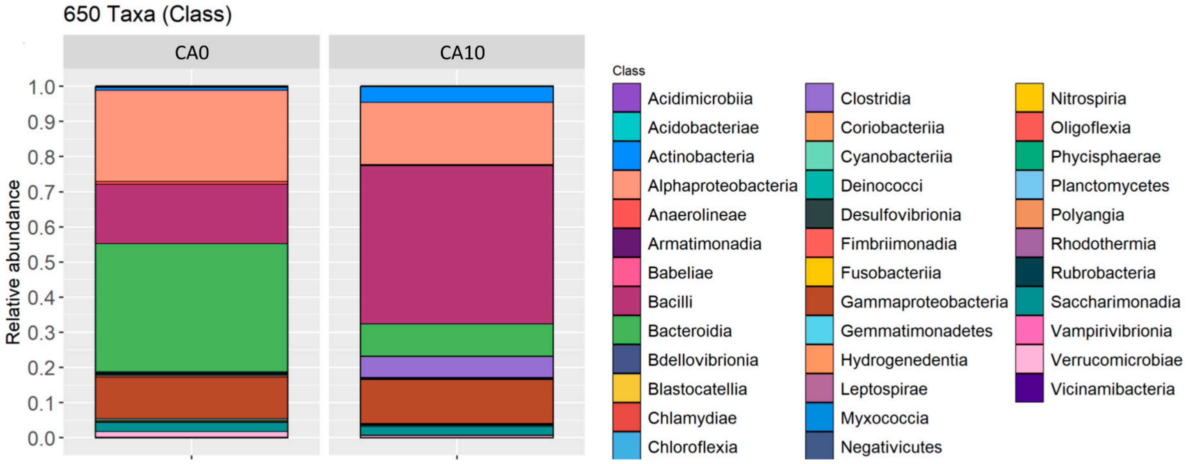

Figure S3 Bacterial characterization in the intestine of prawns was conducted using two groups: one fed with 10 g/kg supplementation food and the non-supplement as the control. The proportions of bacterial taxa at the class level were analyzed in each sample (n = 5). Bar chart inserts were used to illustrate the proportions of bacterial genera present with a relative abundance of at least > 0.2%.

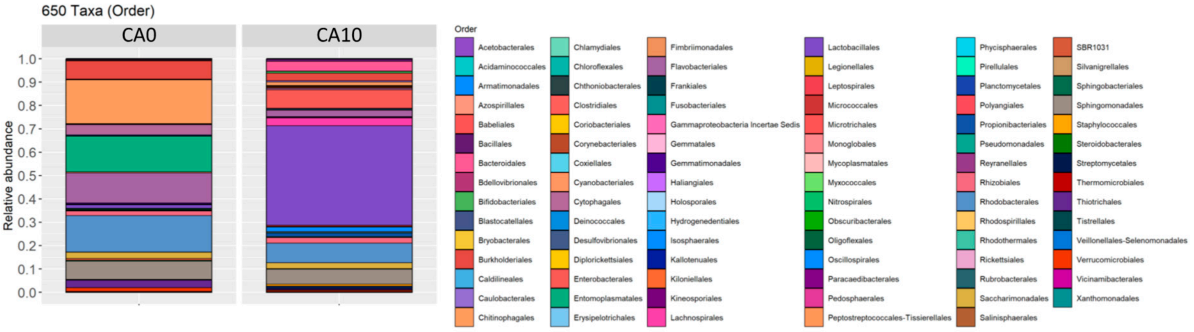

Figure S4 Bacterial characterization in the intestine of prawns was conducted using two groups: one fed with 10 g/kg supplementation food and the non-supplement as the control. The proportions of bacterial taxa at the order level were analyzed in each sample (n = 5). Bar chart inserts were used to illustrate the proportions of bacterial genera present with a relative abundance of at least > 0.2%.

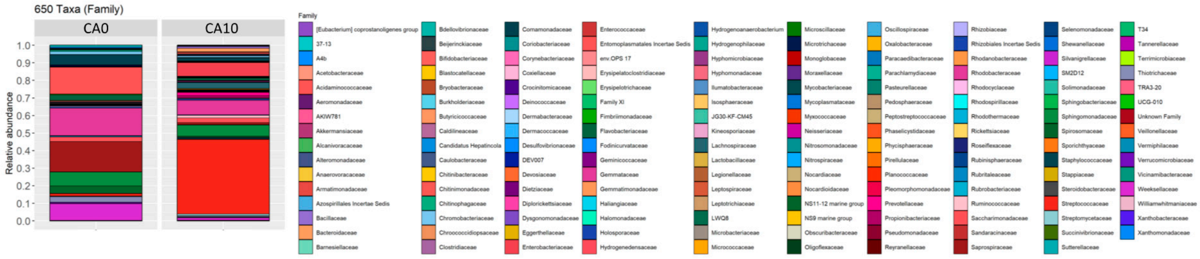

Figure S5 Bacterial characterization in the intestine of prawns was conducted using two groups: one fed with 10 g/kg supplementation food and the non-supplement as the control. The proportions of bacterial taxa at the family level were analyzed in each sample (n = 5). Bar chart inserts were used to illustrate the proportions of bacterial genera present with a relative abundance of at least > 0.2%.
